# Unsupervised cardiometabolic phenotyping unmasks residual MACCE risk beyond LDL-C in acute myocardial infarction after revascularization

**DOI:** 10.3389/fcvm.2026.1869217

**Published:** 2026-07-22

**Authors:** Wendong Xu, Xiaoxu Zhang, Lu Er, Lijie Gu, Ze Zhang, Xiangming Li, Ruoyu Dong, Gexi Cao, Xuechao Wang

**Affiliations:** 1 Department of Cardiology, Hebei Key Laboratory of Precision Medicine Translational Research on Cardiovascular Diseases, Hebei General Hospital, Shijiazhuang, China; 2 Department of Acupuncture and Moxibustion, Hebei General Hospital, Shijiazhuang, China; 3 Department of Ultrasound, Cangzhou Central Hospital, Cangzhou, China; 4 Department of Vascular Surgery, Hebei General Hospital, Shijiazhuang, China; 5 Department of Pharmacy, Hebei General Hospital, Shijiazhuang, China

**Keywords:** acute myocardial infarction, cardiometabolic phenotype, K-means clustering, LDL-C stratification, residual cardiovascular risk, unsupervised clustering

## Abstract

**Background:**

Post-percutaneous coronary intervention (PCI) outcomes in acute myocardial infarction (AMI) remain heterogeneous despite guideline-directed therapy and lower low-density lipoprotein cholesterol (LDL-C) targets. We employed unsupervised clustering of cardiometabolic biomarkers to uncover novel clinical phenotypes that conventional stratifications overlook.

**Methods:**

K-means clustering was applied to 13 standardized cardiometabolic variables in 668 AMI patients who underwent successful PCI. Cluster stability was assessed by bootstrap resampling. Clinical outcomes were evaluated by Kaplan–Meier analysis and multivariable Cox regression. Receiver operating characteristic curves and DeLong testing compared discriminative performance between phenotype classification and LDL-C target attainment.

**Results:**

Three phenotypes were identified: Ph0 Metabolically Balanced (*n* = 391, 58.5%), Ph1 Hyperglycemic-Dyslipidemic (*n* = 107, 16.0%), and Ph2 Decompensated Inflammatory-Catabolic (*n* = 170, 25.4%). Over a median follow-up of 31.9 months, major adverse cardiovascular and cerebrovascular events (MACCE) occurred in 19.4%, 31.8%, and 49.4%, respectively (*P* < 0.001). After full covariate adjustment, Ph2 remained independently associated with MACCE (hazard ratio 2.97, 95% confidence interval 2.00–4.41, *P* < 0.001), while Ph1 was attenuated to non-significance (*P* = 0.136). Despite having the lowest LDL-C, Ph2 carried the highest event rate. LDL-C target attainment (<1.8 mmol/L) did not discriminate MACCE risk [area under the curve (AUC) 0.503], whereas phenotype classification yielded an AUC of 0.626 (DeLong *P* < 0.001).

**Conclusions:**

Unsupervised cardiometabolic phenotyping identified a decompensated inflammatory-catabolic phenotype that carried the highest MACCE risk despite having the lowest LDL-C, representing a high-risk subgroup unrecognizable by conventional lipid-centric stratification. These findings suggest that multi-dimensional metabolic profiling may complement LDL-C targets for residual risk identification in post-PCI AMI patients.

## Introduction

Cardiovascular disease (CVD) remains the leading cause of death and disability globally (1). The 2023 World Heart Report documented 20.5 million CVD-related deaths in 2021, of which acute myocardial infarction (AMI) accounted for the largest proportion (2). Despite early percutaneous coronary intervention (PCI) with guideline-directed medical therapy now constituting the standard of care for AMI, major adverse cardiovascular and cerebrovascular events (MACCE) rates following PCI for AMI remain substantial, ranging from 10% to 20% within one to two years in real-world populations (3–5).

While low-density lipoprotein cholesterol (LDL-C) is traditionally recognized as a central driver of atherosclerotic progression and a cornerstone of lipid-lowering strategies (6), its prognostic significance in the post-AMI setting remains a subject of considerable debate. In a Korean cohort of 9,571 MI patients (2005–2008) (7), admission LDL-C showed no association with 1-year mortality after adjusting for comorbidities, PCI-related factors, and medication use. In contrast, a U.S. single-center study of 7,071 AMI patients (2001–2011) found that hypercholesterolemia was actually linked to lower 5-year mortality, even after propensity score matching and Cox regression (8). To explain these conflicting findings, researchers have investigated various non-lipid factors that may interfere with the prognostic value of LDL-C. For instance, large-scale evidence suggests that this inverse relationship is primarily manifested in patients with systemic inflammation [high-sensitivity C-reactive protein (hsCRP) ≥ 3 mg/L], indicating that inflammatory burden may supersede lipid levels as a primary risk driver (9). Also, in malnourished patients, low LDL-C often reflects poor nutritional status and protein-calorie loss rather than good lipid control (10). Today, the early use of high-intensity statins and PCSK9 inhibitors further complicates the situation, as admission LDL-C may simply show the effect of these drugs rather than the patient's true baseline risk. Therefore, managing LDL-C alone in patients with AMI has limitations.

Beyond lipid levels, the prognosis after AMI is driven by multidimensional cardiometabolic factors. Nutritional status, often measured by serum albumin, is an underappreciated predictor; a meta-analysis of 21,667 patients found that low albumin independently doubles the risk of all-cause mortality (11). Similarly, systemic inflammation plays a role that lipid levels cannot capture. High C-reactive protein (CRP) has been confirmed as an independent risk factor for cardiovascular death and MACCE in over 18,000 AMI patients treated with PCI (12). Cardiac function at the time of injury also provides critical information. For instance, baseline NT-proBNP was one of the strongest predictors of death and heart failure in the PARADISE-MI trial (13). Glycaemia dysregulation represents another dimension of prognostic risk that lipid measurements cannot capture. A systematic review and meta-analysis of 66 studies demonstrated that both admission fasting blood glucose and HbA1c are independently associated with adverse outcomes after ST-elevation myocardial infarction, with admission hyperglycemia associated with a nearly twofold increase in composite MACCE (pooled RR: 1.99, 95% CI: 1.54–2.58) and HbA1c independently predicting long-term mortality (14). Renal function, as reflected by serum creatinine or estimated glomerular filtration rate, is an established independent predictor of mortality and heart failure after AMI, and its inclusion in risk models substantially improves prognostic discrimination (15). Additionally, the Killip classification remains a reliable tool for assessing hemodynamic stability at presentation (16). Ultimately, no single biomarker can fully capture the complex heterogeneity of AMI patients.

Data-driven unsupervised phenotyping is a useful way to handle this complexity. By using clustering algorithms, we can identify natural subgroups of patients with different biological profiles and outcomes without relying on pre-set assumptions. This paradigm has demonstrated clinical value in cardiovascular medicine, such as identifying distinct groups in cardiogenic shock (17) and STEMI populations (18). In these cases, metabolic factors often prove to be more important for classification than simple anatomical variables. However, no study has yet integrated lipid, glycemic, inflammatory, nutritional, and cardiac decompensation dimensions to characterize the specific phenotypes of AMI patients receiving PCI.

In this study, we applied unsupervised K-means clustering to 668 consecutive AMI patients who underwent successful PCI and received guideline-directed therapy, integrating lipid, glycemic, inflammatory, nutritional, and cardiac decompensation variables to characterize cardiometabolic phenotypes. We aimed to test whether unsupervised clustering of multidimensional cardiometabolic biomarkers could identify phenotypic subgroups with distinct residual risk profiles in this optimally treated cohort, and whether such phenotypic classification captures prognostic heterogeneity that LDL-C—as the predominant therapeutic target—does not reflect. This study was designed to characterize cardiometabolic phenotypes rather than to develop an individual-level prediction model.

## Methods

### Study design and population

This was a retrospective cohort study. The cohort consisted of consecutive patients with AMI who underwent successful PCI at Hebei General Hospital between December 2020 and January 2024. AMI was defined based on the Fourth Universal Definition, including both STEMI and NSTEMI. We included patients aged between 18 and 96 years with a confirmed diagnosis of AMI and successful PCI. Patients were excluded based on the following criteria: (1) Repeated hospitalization; (2) PCI failure (residual stenosis ≥ 20%–30% or TIMI flow < 3); (3) severe hepatic and renal impairment, including renal failure requiring maintenance dialysis; (4) malignant tumors or autoimmune diseases; (5) active infectious diseases, including COVID-19; (6) A family history of hypertriglyceridemia; (7) patients undergoing PCI supported by extracorporeal membrane oxygenation (ECMO) or other mechanical circulatory support (MCS) devices; (8) significant missing clinical data; (9) loss to follow-up. The study was approved by the Institutional Review Board of Hebei General Hospital (Approval No. 2026-LW-098). The requirement for individual informed consent was waived given the retrospective nature of the study. This study was conducted and reported in accordance with the Strengthening the Reporting of Observational Studies in Epidemiology (STROBE) guidelines.

### Data collection and follow-up

Baseline clinical data were extracted from electronic medical records, including demographic characteristics, medical history, laboratory examination obtained from blood samples drawn within 24 h of admission, echocardiographic parameters, operative records, and discharge medications. Follow-up commenced from the date of hospital admission and continued until the first occurrence of an endpoint event or the administrative censoring date of December 31, 2025. Follow-up data were obtained through outpatient clinic records, telephone interviews, and review of electronic medical records.

### Clinical endpoints

The primary endpoint was major adverse cardiovascular and cerebrovascular events (MACCE), defined as a composite of cardiovascular death, non-fatal myocardial infarction, non-fatal stroke, unplanned revascularization, hospitalization for heart failure, and hospitalization for unstable angina. The secondary endpoint was all-cause death.

### Clustering variables and data preprocessing

Clustering variables were selected based on four core criteria: a established biological relevance to AMI prognosis; multidimensional representation across independent cardiometabolic domains; the minimization of collinearity to ensure informational independence; and clinical feasibility, requiring all indicators to be part of routine admission testing. Based on the above principles, 13 variables were ultimately included, covering five biological dimensions: lipid indices [LDL-C, triglycerides, HDL-C, lipoprotein(a)], glycemic markers (fasting glucose, HbA1c), inflammatory and nutritional parameters (CRP, albumin, hemoglobin), and indices of cardiac decompensation (LVEF, NT-proBNP, Killip class, creatinine). Variables with high collinearity or redundant clinical information were excluded to maintain analytical parsimony. Missing data were handled according to the proportion of missingness: variables with less than 10% missing values were imputed using median substitution, whereas HbA1c (16.5% missing) were imputed using multivariate imputation by chained equations (MICE). Missing values for NT-proBNP (9.4%) were addressed via multiple imputation by chained equations (MICE). This approach was preferred over simple median substitution to better accommodate the variable's right-skewed distribution and its subsequent requirement for log-transformation.HbA1c missing values were significantly more frequent in non-diabetic patients, suggesting a missing-not-at-random pattern; this was accounted for by including diabetes status as a predictor variable in the MICE model. NT-proBNP was log₁₀-transformed prior to analysis to correct for right-skewed distribution. All variables were winsorized at the 1st and 99th percentiles to mitigate the influence of extreme outliers, followed by Z-score standardization.

### Clustering analysis and optimal cluster selection

Unsupervised K-means clustering was applied to the preprocessed and standardized 13-variable matrix. The optimal number of clusters was determined by evaluating K = 2 through K = 6 using four validity indices: the Silhouette coefficient, Calinski-Harabasz index, Davies-Bouldin index, and Gap statistic. The final solution was selected based on the combination of statistical validity and clinical interpretability. K-means was run with 100 random initialisations and a maximum of 500 iterations per run, with a fixed random seed to ensure reproducibility.

### Cluster stability validation

Clustering stability was assessed through 200 bootstrap resamples, with the Silhouette coefficient computed for each iteration to derive a 95% confidence interval. Algorithm robustness was further evaluated by comparing K-means assignments with those from a Gaussian Mixture Model (GMM) and Partitioning Around Medoids (PAM), with agreement quantified using the Adjusted Rand Index (ARI).

### Statistical analysis

All statistical analyses were performed using Python (version 3.11) and R (version 4.5.2). A two-sided *P* < 0.05 was considered statistically significant. For continuous variables, distributional normality was assessed using the Shapiro–Wilk test, supplemented by evaluation of skewness and kurtosis. Normality was strictly defined by the concurrence of a Shapiro–Wilk *P* > 0.05, absolute skewness < 1, and absolute kurtosis < 3. Normally distributed data are expressed as mean ± standard deviation (SD) and compared using one-way ANOVA. Non-normally distributed variables are reported as median [interquartile range (IQR)] and compared using the Kruskal–Wallis test. *post-hoc* pairwise comparisons were conducted using the Wilcoxon rank-sum test, with *P* values adjusted via the Bonferroni method (original *P* values multiplied by 3). Categorical data are presented as frequencies (percentages) and compared using Pearson's chi-square test or Fisher's exact test, as appropriate. Cumulative incidence of MACCE and all-cause death across phenotypes was estimated using Kaplan–Meier analysis. Overall between-group differences were assessed by the log-rank test, and pairwise comparisons were performed with Bonferroni correction. To evaluate the primary MACCE endpoint, three pre-specified nested Cox proportional hazards models were constructed, using Phenotype 0 as the reference group. Model 1 adjusted for age, sex, and BMI; Model 2 further accounted for smoking, hypertension, diabetes, dyslipidemia, and relevant medical history (prior MI, revascularization, stroke, and CKD); and Model 3—the fully adjusted model—additionally included procedural and clinical severity markers (STEMI, multivessel disease, implantation of ≥2 stents, and admission GRACE score) along with discharge medications (beta-blockers and PCSK9 inhibitors). Variables comprising the clustering input—including Killip classification, serum creatinine, LVEF, NT-proBNP, CRP, serum albumin, hemoglobin, and lipid parameters—were intentionally excluded from all Cox models to avoid overadjustment bias. Statin therapy was additionally excluded owing to near-universal use in this cohort (99.0%), which precluded meaningful statistical adjustment. The proportional hazards assumption was verified for all Cox models using Schoenfeld residual tests; no significant violations were detected (all individual and global *P* > 0.05).Results are expressed as hazard ratios (HR) with 95% confidence intervals (CI). To further evaluate heterogeneity in clinical outcomes, individual MACCE components—including cardiovascular death, unplanned revascularization, and hospitalisation for heart failure—were analysed separately using Kaplan–Meier curves and log-rank testing. Subgroup analyses were performed by stratifying patients according to diabetes status and AMI type (STEMI vs. NSTEMI), with Cox proportional hazards regression repeated within each stratum using the same three-model adjustment sequence. Sensitivity analyses were conducted to ensure that the imputation of HbA1c did not bias the primary outcomes.

## Results

### Identification and characterization of cardiometabolic phenotypes

**Figure 1:**
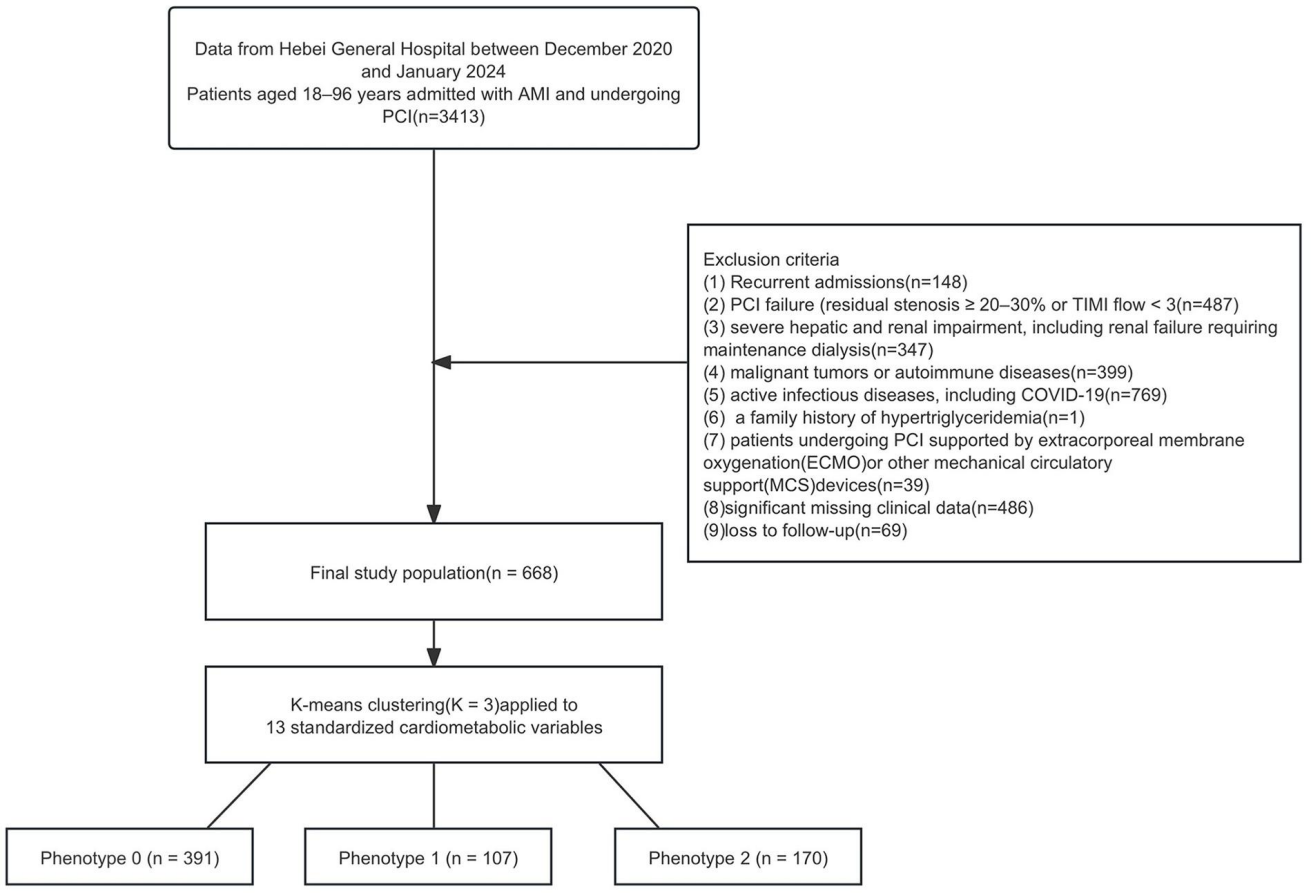
Patient selection and study flowchart. AMI, acute myocardial infarction; ECMO, extracorporeal membrane oxygenation; MCS, mechanical circulatory support; PCI, percutaneous coronary intervention; TIMI, Thrombolysis in Myocardial Infarction.

**Figure 2:**
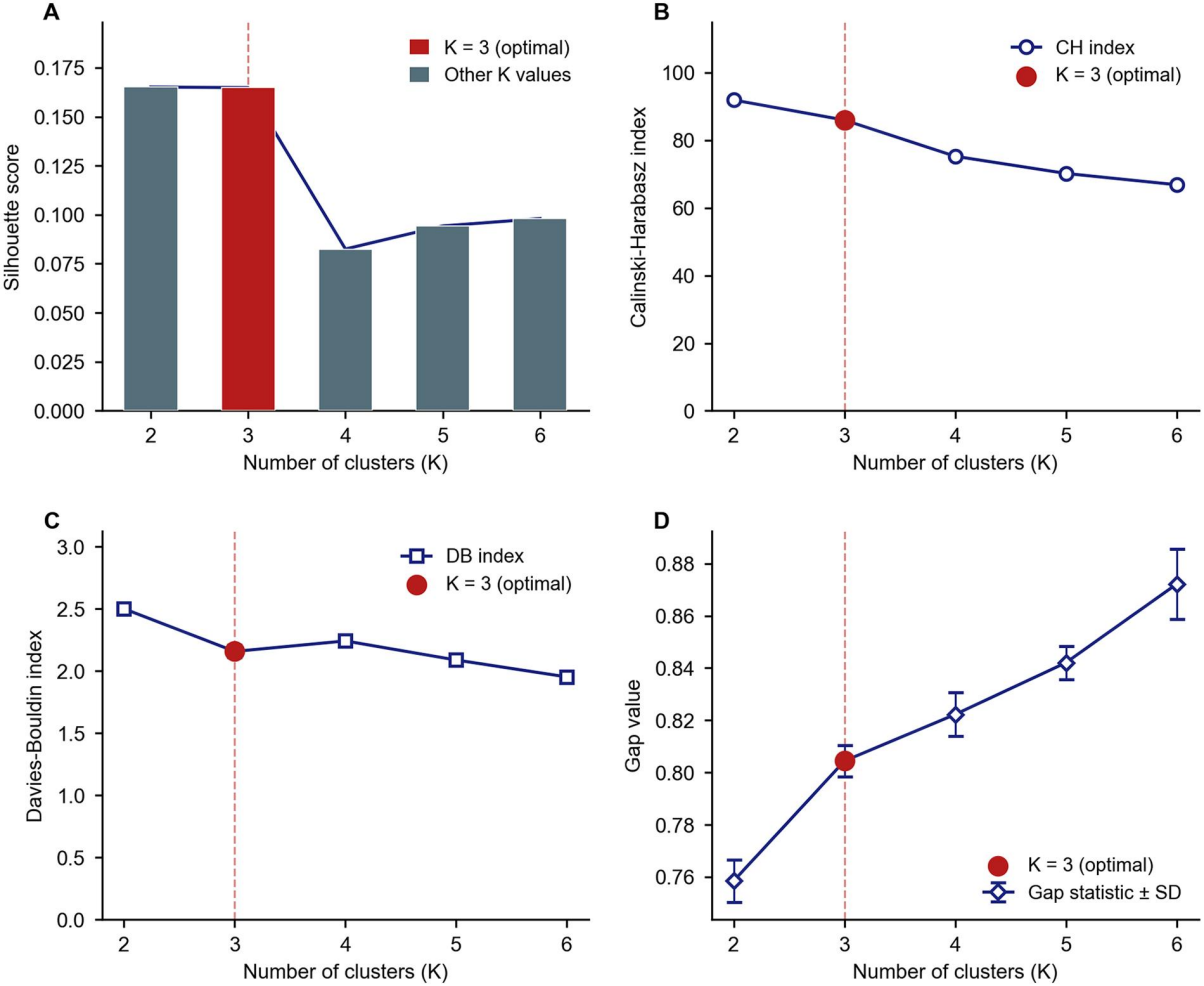
Determination of optimal cluster number using four validity indices. Selection of optimal cluster number (K = 2–6) using four internal validity indices. **(A)** Silhouette score; **(B)** Calinski-Harabasz index; **(C)** Davies-Bouldin index; **(D)** Gap statistic. Dashed vertical lines and red markers indicate the selected solution of K = 3.

A total of 668 post-PCI AMI patients were included (Figure 1). K-means clustering was applied to 13 standardized cardiometabolic variables, encompassing lipid indices [LDL-C, triglycerides, HDL-C, lipoprotein(a)], glycemic markers (fasting glucose, HbA1c), inflammatory and nutritional parameters (CRP, albumin, hemoglobin), and indices of cardiac decompensation (LVEF, NT-proBNP, Killip class, creatinine). Cluster solutions ranging from K = 2 to K = 6 were evaluated against four internal validity indices (supplementary Table S1; Figure 2). Although K = 2 yielded the highest Silhouette score (0.1651), the difference from K = 3 was negligible (0.1648, *Δ* < 0.001). In contrast, K = 3 demonstrated a substantially superior Davies-Bouldin index (2.158 vs. 2.501), indicating better-defined cluster separation, while the Calinski-Harabasz index and gap statistic both showed meaningful improvement from K = 2 to K = 3 before attenuating. Solutions with K ≥ 4 showed declining cohesion without meaningful improvement in separation. Based on these metrics and greater clinical interpretability, K = 3 was selected as the optimal solution.

**Figure 3:**
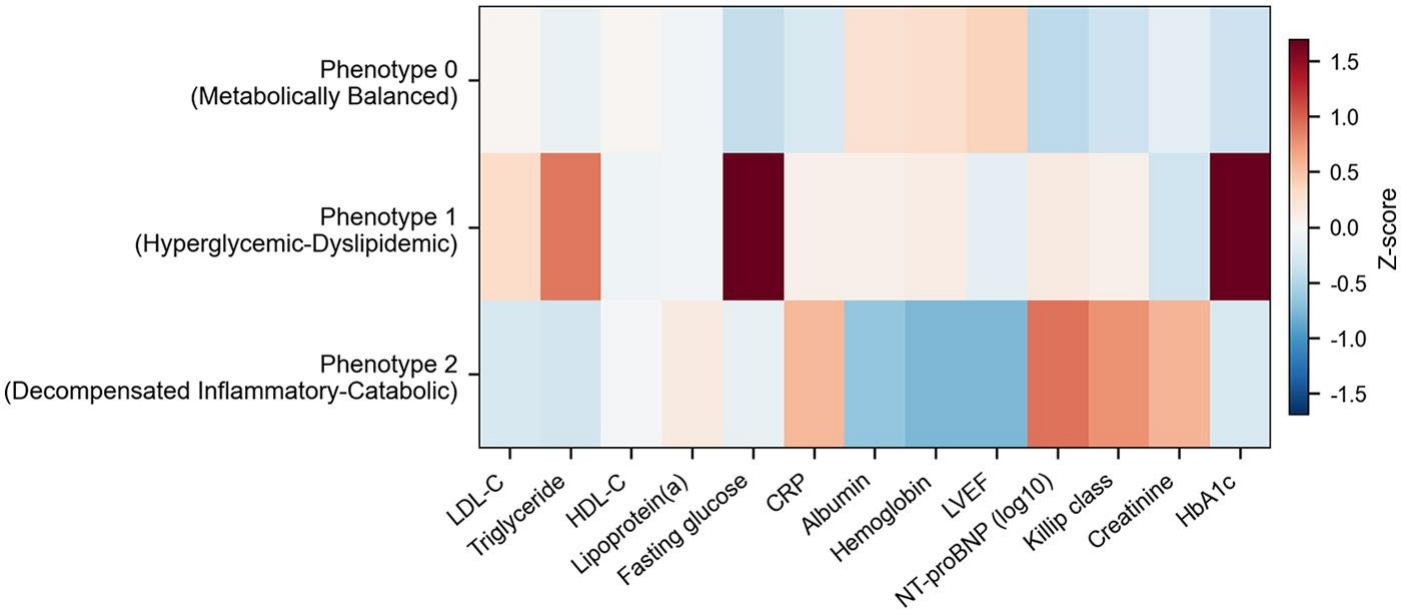
Standardized variable profiles across three cardiometabolic phenotypes. Heatmap of standardized variable means (Z-scores). Red indicates values above the cohort mean; blue indicates values below. CRP, C-reactive protein; HbA1c, glycated hemoglobin; HDL-C, high-density lipoprotein cholesterol; LDL-C, low-density lipoprotein cholesterol; LVEF, left ventricular ejection fraction; NT-proBNP, N-terminal pro-B-type natriuretic peptide.

**Figure 4:**
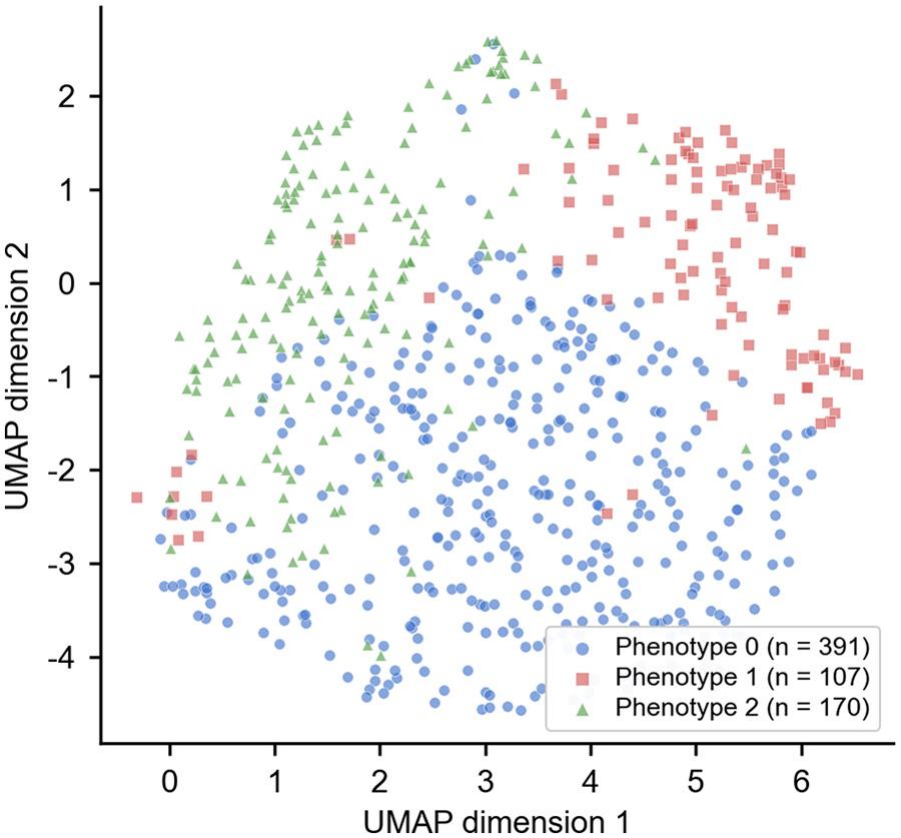
UMAP projection of cardiometabolic clustering variables. Each point represents one patient (*n* = 668). Colours and marker shapes indicate phenotype assignment. UMAP parameters: n_neighbours = 30, min_dist = 0.3, Euclidean metric. UMAP, uniform manifold approximation and projection.

Phenotype labels were assigned *post hoc* according to the dominant standardized variable profiles observed in the heatmap of cluster centroids (Figure 3; detailed characteristics are provided in supplementary Table S2). UMAP projection confirmed partial spatial separation among the three phenotypes, with Phenotype 1 clustering predominantly in the upper-right region and Phenotype 2 occupying the upper-left, while Phenotype 0 was more broadly distributed across the lower portion of the embedding space (Figure 4).

Cluster stability was confirmed by bootstrap resampling across 200 iterations, yielding a mean Silhouette score of 0.1694 (95% CI: 0.1376–0.2081; supplementary Figure S1), supporting the robustness of the three-cluster structure. To further assess algorithmic robustness, K-means assignments were compared with two alternative clustering methods. Agreement with Partitioning Around Medoids (PAM) was near-perfect (ARI = 0.997), indicating that the three-phenotype structure was essentially identical across the two hard-clustering approaches. Agreement with the Gaussian Mixture Model (GMM) was moderate (ARI = 0.308), likely reflecting the inherent methodological difference between hard and soft clustering rather than structural instability, consistent with the lower Silhouette score observed for GMM (0.1106 vs. 0.1648 for K-means; Table 1).

**Table 1:** Clustering stability and algorithm consistency validation.

Validation method	Metric	Value	Interpretation
Bootstrap (*n* = 200)	Silhouette mean ± SD	0.1694 ± 0.0176	Internal stability
Bootstrap (*n* = 200)	95% CI	0.1376–0.2081	Narrow CI indicates stable clustering
Algorithm consistency	K-means Silhouette	0.1648	Primary algorithm
Algorithm consistency	GMM Silhouette	0.1106	Alternative algorithm
Algorithm consistency	K-means (seed=99)	0.1648	Reproducible result
Algorithm consistency	K-means vs. GMM ARI	0.308	Moderate agreement
Algorithm consistency	K-means vs. PAM ARI	0.997	Near-perfect agreement

ARI, adjusted Rand index; CI, confidence interval; GMM, Gaussian mixture model; PAM, partitioning around medoids. Bootstrap resampling (*n* = 200) with replacement was performed to assess internal stability. Algorithm consistency was evaluated by comparing K-means with GMM and PAM.

The defining characteristics of each phenotype are summarized below: Phenotype 0: Metabolically Balanced (*n* = 391, 58.5%; supplementary Table S2) Patients in this cluster exhibited relatively preserved lipid, glycemic, and hemodynamic profiles, with Z-scores across all 13 variables remaining close to the cohort mean. This group constituted the largest proportion of the study population and served as the clinical reference for inter-phenotype comparisons. Phenotype 1: Hyperglycemic-Dyslipidemic (*n* = 107, 16.0%; supplementary Table S2) This phenotype was primarily defined by marked elevations in fasting glucose (Z =  + 1.69), HbA1c (Z =  + 1.68), and triglycerides (Z =  + 0.89), indicating a phenotype driven predominantly by poor glycemic control and atherogenic dyslipidemia. Cardiac and inflammatory indices remained comparatively unremarkable in this group. Phenotype 2: Decompensated Inflammatory-Catabolic (*n* = 170, 25.4%; supplementary Table S2) This phenotype was characterized by concurrent elevations in inflammatory and catabolic markers, impaired cardiac function, and multiorgan involvement. CRP was elevated (Z = +0.55), while albumin (Z = −0.66) and hemoglobin (Z = −0.76) were reduced. Cardiac decompensation was reflected by depressed LVEF (Z = −0.77) and elevated NT-proBNP (Z = +0.92), with Killip class III–IV present in 20.6% of patients, substantially higher than in Phenotype 1 (3.7%) and Phenotype 0 (0.6%; Z = +0.76). Creatinine was also elevated (Z = +0.57), indicating concurrent renal impairment. Inter-group comparisons confirmed significant differences across all 13 clustering variables (all *P* < 0.05). Notably, LDL-C Z-scores were broadly comparable across phenotypes (Ph0: +0.03; Ph1: +0.31; Ph2: −0.27), indicating that lipid-based differentiation did not drive cluster separation. Detailed standardized profiles and raw values are provided in supplementary Table S2.

### Baseline characteristics

Baseline characteristics are presented in Table 2, with statistically significant inter-group differences across most clinical and biochemical variables. The three phenotypes differed substantially in demographic profile. Ph2 patients were markedly older (median 73.5 years) and had the lowest proportion of male sex (54.7%), compared with Ph0 (61.0 years, 85.9% male) and Ph1 (60.0 years, 65.4% male; both *P* < 0.001). Ph2 also carried the highest comorbidity burden, including the highest prevalence of hypertension (74.7%), prior MI (15.3%), and CKD (22.9% vs. 4.1% in Ph0), alongside the highest GRACE score (median 180.5) and most pronounced markers of cardiac decompensation: lowest LVEF (median 48.5%), highest NT-proBNP, and the greatest proportion of Killip class III–IV (20.6% vs. 0.6% in Ph0). Ph1 was distinguished by the most severe metabolic derangement, with the highest HbA1c, fasting glucose, triglycerides, LDL-C, and apolipoprotein B, and the greatest prevalence of multivessel disease (70.1%). Pre-procedural TIMI flow distribution and culprit vessel involvement were comparable across phenotypes, and all patients achieved TIMI Grade 3 flow post-PCI. Discharge pharmacotherapy reflected phenotype-specific risk profiles: Ph2 received more diuretics (76.5%), angiotensin receptor-neprilysin inhibitors (ARNI), and mineralocorticoid receptor antagonists (MRA), while Ph1 received more sodium-glucose cotransporter-2 (SGLT2) inhibitors (54.2%) and insulin (42.1%).

**Table 2:** Baseline characteristics of patients stratified by cardiometabolic phenotype.

Characteristic	Overall (*n* = 668)	Ph0 (*n* = 391)	Ph1 (*n* = 107)	Ph2 (*n* = 170)	P
n	668	391	107	170	
Age	63.00 [55.00, 73.00]	61.00 [53.50, 70.00]	60.00 [53.00, 69.00]	73.50 [65.00, 80.00]	<0.001
BMI	25.37 [23.39, 27.38]	25.71 [23.84, 27.68]	24.77 [22.65, 26.69]	25.14 [22.49, 27.09]	0.009
SBP, mmHg	135.55 (24.44)	135.68 (22.74)	139.06 (24.38)	133.05 (27.90)	0.137
Heart rate, bpm	78.00 [67.00, 89.00]	75.00 [66.00, 84.00]	85.00 [77.00, 98.00]	80.00 [71.00, 96.00]	<0.001
Hemoglobin, g/L	137.00 [124.00, 149.00]	142.00 [131.00, 153.00]	139.00 [126.50, 151.50]	121.00 [107.75, 135.00]	<0.001
Platelet, ×10⁹/L	228.00 [190.00, 270.75]	228.00 [196.00, 266.00]	230.50 [189.75, 284.25]	227.00 [178.50, 274.50]	0.453
White blood cell, ×10⁹/L	8.87 [7.13, 11.03]	8.70 [6.96, 10.68]	9.14 [7.90, 11.07]	9.02 [7.35, 11.49]	0.079
hs-cTnT, log10 (ng/L)	3.30 [2.97, 3.30]	3.30 [2.91, 3.30]	3.30 [2.89, 3.30]	3.30 [3.20, 3.30]	0.001
HbA1c, %	6.15 [5.70, 7.40]	5.90 [5.50, 6.40]	8.60 [7.68, 9.95]	6.00 [5.60, 6.70]	<0.001
Fasting glucose, mmol/L	6.06 [4.99, 7.95]	5.54 [4.83, 6.54]	12.47 [10.03, 14.68]	5.97 [5.02, 7.53]	<0.001
Total cholesterol, mmol/L	4.70 [3.92, 5.50]	4.72 [4.02, 5.50]	5.17 [4.52, 5.96]	4.18 [3.56, 5.04]	<0.001
Triglyceride, mmol/L	1.37 [0.94, 1.99]	1.37 [0.90, 1.94]	2.21 [1.30, 3.20]	1.18 [0.89, 1.56]	<0.001
LDL-C, mmol/L	3.02 [2.46, 3.65]	3.06 [2.53, 3.65]	3.26 [2.79, 3.87]	2.70 [2.21, 3.28]	<0.001
HDL-C, mmol/L	1.06 [0.92, 1.25]	1.08 [0.93, 1.26]	1.07 [0.83, 1.23]	1.03 [0.90, 1.23]	0.369
Apolipoprotein B, g/L	0.92 [0.73, 1.16]	0.94 [0.74, 1.15]	1.11 [0.92, 1.31]	0.80 [0.66, 1.01]	<0.001
Lipoprotein(a), mg/L	174.60 [84.75, 342.05]	167.10 [83.73, 333.25]	164.10 [70.75, 298.70]	217.95 [109.88, 399.72]	0.017
CRP, mg/L	3.53 [0.82, 13.36]	2.16 [0.52, 6.22]	5.82 [2.32, 16.75]	10.32 [2.97, 35.63]	<0.001
Albumin, g/L	38.70 [36.40, 40.90]	39.60 [37.35, 41.65]	38.60 [36.80, 41.55]	36.70 [34.20, 38.70]	<0.001
Creatinine, µmol/L	70.40 [61.65, 83.25]	69.35 [62.28, 78.77]	63.20 [53.35, 72.35]	81.10 [67.55, 97.80]	<0.001
LVEF, %	57.00 [50.00, 61.00]	59.00 [55.00, 62.00]	54.00 [50.00, 58.00]	48.50 [42.00, 54.75]	<0.001
NT-proBNP, log10 (pg/mL)	2.93 [2.66, 3.26]	2.76 [2.50, 2.98]	3.03 [2.80, 3.31]	3.39 [3.14, 3.69]	<0.001
Admission GRACE score	154.00 [133.75, 174.25]	144.00 [128.50, 160.50]	149.00 [129.50, 168.00]	180.50 [163.00, 205.25]	<0.001
Male sex (%)	499 (74.7)	336 (85.9)	70 (65.4)	93 (54.7)	<0.001
Smoking (%)	446 (66.8)	296 (75.7)	62 (57.9)	88 (51.8)	<0.001
Diabetes mellitus (%)	216 (32.3)	73 (18.7)	99 (92.5)	44 (25.9)	<0.001
Hypertension (%)	398 (59.6)	202 (51.7)	69 (64.5)	127 (74.7)	<0.001
Dyslipidemia (%)	54 (8.1)	31 (7.9)	12 (11.2)	11 (6.5)	0.364
Prior MI (%)	61 (9.1)	25 (6.4)	10 (9.3)	26 (15.3)	0.003
Prior revascularization (%)	61 (9.1)	32 (8.2)	9 (8.4)	20 (11.8)	0.385
Prior stroke (%)	75 (11.2)	38 (9.7)	11 (10.3)	26 (15.3)	0.149
CKD (%)	60 (9.0)	16 (4.1)	5 (4.7)	39 (22.9)	<0.001
STEMI (%)	484 (72.5)	284 (72.6)	86 (80.4)	114 (67.1)	0.054
Multivessel disease (%)	344 (51.5)	177 (45.3)	75 (70.1)	92 (54.1)	<0.001
Implantation of ≥2 stents (%)	161 (24.1)	83 (21.3)	26 (24.3)	52 (30.6)	0.061
Drug-coated balloon (%)					0.528
None	584 (87.4)	338 (86.4)	91 (85.0)	155 (91.2)	
1 vessel	73 (10.9)	46 (11.8)	13 (12.1)	14 (8.2)	
2 vessels	10 (1.5)	6 (1.5)	3 (2.8)	1 (0.6)	
3 vessels	1 (0.1)	1 (0.3)	0 (0.0)	0 (0.0)	
Killip class (%)					<0.001
Killip I	304 (45.5)	240 (61.4)	39 (36.4)	25 (14.7)	
Killip II	323 (48.4)	149 (38.1)	64 (59.8)	110 (64.7)	
Killip III	13 (1.9)	1 (0.3)	0 (0.0)	12 (7.1)	
Killip IV	28 (4.2)	1 (0.3)	4 (3.7)	23 (13.5)	
Pre-procedural TIMI flow grade (%)					0.761
Grade 0	277 (41.5)	153 (39.1)	50 (46.7)	74 (43.5)	
Grade 1	40 (6.0)	23 (5.9)	5 (4.7)	12 (7.1)	
Grade 2	59 (8.8)	35 (9.0)	10 (9.3)	14 (8.2)	
Grade 3	292 (43.7)	180 (46.0)	42 (39.3)	70 (41.2)	
Culprit: LAD (%)	323 (48.4)	193 (49.4)	48 (44.9)	82 (48.2)	0.711
Culprit: LCX (%)	87 (13.0)	54 (13.8)	15 (14.0)	18 (10.6)	0.55
Culprit: RCA (%)	258 (38.6)	146 (37.3)	44 (41.1)	68 (40.0)	0.709
Culprit: LM (%)	19 (2.8)	7 (1.8)	2 (1.9)	10 (5.9)	0.029
Aspirin (%)	658 (98.5)	386 (98.7)	107 (100.0)	165 (97.1)	0.125
Clopidogrel (%)	177 (26.5)	79 (20.2)	26 (24.3)	72 (42.4)	<0.001
Ticagrelor (%)	553 (82.8)	343 (87.7)	94 (87.9)	116 (68.2)	<0.001
Statins (%)	661 (99.0)	388 (99.2)	104 (97.2)	169 (99.4)	0.148
Beta-blockers (%)	605 (90.6)	351 (89.8)	99 (92.5)	155 (91.2)	0.656
PCSK9 inhibitors (%)	221 (33.1)	130 (33.2)	53 (49.5)	38 (22.4)	<0.001
ACEI/ARB (%)	287 (43.0)	185 (47.3)	54 (50.5)	48 (28.2)	<0.001
ARNI (%)	166 (24.9)	79 (20.2)	31 (29.0)	56 (32.9)	0.003
MRA (%)	494 (74.0)	268 (68.5)	86 (80.4)	140 (82.4)	0.001
Diuretics (%)	296 (44.3)	113 (28.9)	53 (49.5)	130 (76.5)	<0.001
SGLT2 inhibitors (%)	141 (21.1)	48 (12.3)	58 (54.2)	35 (20.6)	<0.001
Insulin (%)	88 (13.2)	24 (6.1)	45 (42.1)	19 (11.2)	<0.001

BMI, body mass index; SBP, systolic blood pressure; hs-cTnT, high-sensitivity cardiac troponin T; HbA1c, glycated hemoglobin; LDL-C, low-density lipoprotein cholesterol; HDL-C, high-density lipoprotein cholesterol; ApoB, apolipoprotein B; NT-proBNP, N-terminal pro-B-type natriuretic peptide; CRP, C-reactive protein; LVEF, left ventricular ejection fraction; TIMI, Thrombolysis In Myocardial Infarction; STEMI, ST-elevation myocardial infarction; ACEI, angiotensin-converting enzyme inhibitor; ARB, angiotensin receptor blocker; ARNI, angiotensin receptor-neprilysin inhibitor; MRA, mineralocorticoid receptor antagonist; SGLT2, sodium-glucose cotransporter-2; PCSK9, proprotein convertase subtilisin/kexin type 9; Ph0, Phenotype 0 (Metabolically Balanced); Ph1, Phenotype 1 (Hyperglycemic-Dyslipidemic); Ph2, Phenotype 2 (Decompensated Inflammatory-Catabolic).

Data are presented as *n* (%) for categorical variables, median [interquartile range] for non-normally distributed continuous variables, or mean (SD) for normally distributed continuous variables. *P* values were calculated using Chi-square test or Fisher's exact test for categorical variables, Kruskal–Wallis test for non-normally distributed continuous variables, or ANOVA for normally distributed continuous variables.

### Clinical outcomes across cardiometabolic phenotypes

**Figure 5:**
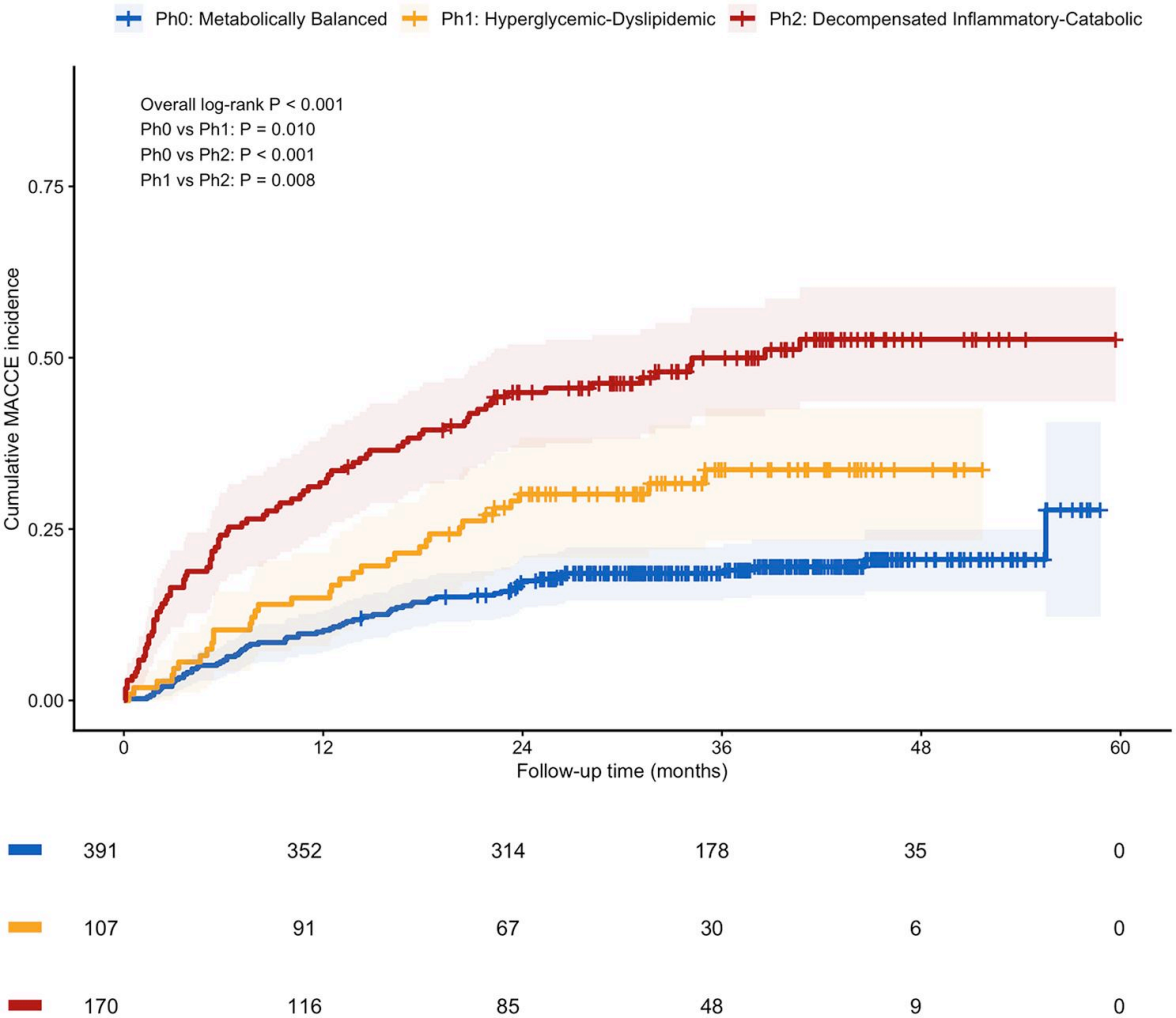
Cumulative MACCE incidence by cardiometabolic phenotype. Kaplan–Meier curves with 95% confidence intervals. Numbers at risk are shown below the *x*-axis. *P* values from log-rank test with Bonferroni correction for pairwise comparisons. MACCE, major adverse cardiovascular and cerebrovascular events.

**Figure 6:**
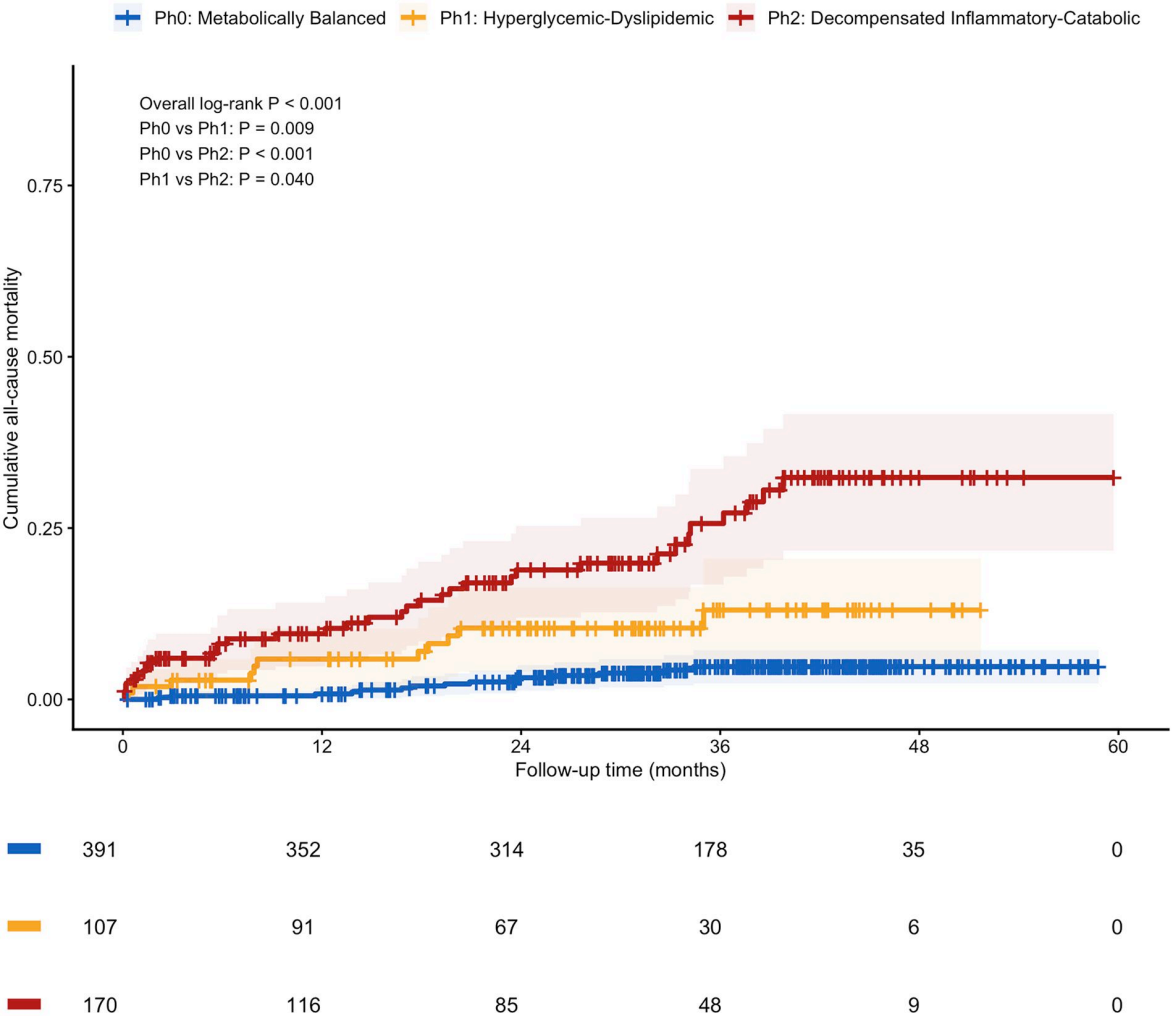
Cumulative all-cause mortality by cardiometabolic phenotype. Kaplan–Meier curves with 95% confidence intervals. Numbers at risk are shown below the *x*-axis. *P* values from log-rank test with Bonferroni correction for pairwise comparisons.

Over a median follow-up of 31.9 months (IQR: 21.6–41.1 months), MACCE occurred in 194 patients (29.0%), with a graded increase in cumulative incidence across phenotypes: 19.4% in Ph0, 31.8% in Ph1, and 49.4% in Ph2 (overall log-rank *P* < 0.001; Ph0 vs. Ph1: *P* = 0.010; Ph0 vs. Ph2: *P* < 0.001; Ph1 vs. Ph2: *P* = 0.008; Table 3; Figure 5). All-cause mortality exhibited a similar gradient, with cumulative incidences of 3.8%, 10.3%, and 20.6% in Ph0, Ph1, and Ph2, respectively (overall log-rank *P* < 0.001; Ph0 vs. Ph1: *P* = 0.009; Ph0 vs. Ph2: *P* < 0.001; Ph1 vs. Ph2: *P* = 0.040; Table 3; Figure 6).

**Table 3:** Clinical outcomes and event rates across three cardiometabolic phenotypes.

Endpoint	Ph0 (*n* = 391)	Ph1 (*n* = 107)	Ph2 (*n* = 170)	Overall P	Ph0 vs. Ph1	Ph0 vs. Ph2	Ph1 vs. Ph2
MACCE	76 (19.4)	34 (31.8)	84 (49.4)	<0.001	0.010	<0.001	0.008
All-cause death	15 (3.8)	11 (10.3)	35 (20.6)	<0.001	0.009	<0.001	0.040
CV death	9 (2.3)	8 (7.5)	22 (12.9)	<0.001	0.016	<0.001	—
Non-fatal MI	16 (4.1)	8 (7.5)	26 (15.3)	<0.001	0.194	<0.001	—
Non-fatal stroke	3 (0.8)	3 (2.8)	4 (2.4)	0.089	0.222	0.139	—
Unplanned revascularization	30 (7.7)	9 (8.4)	15 (8.8)	0.373	1.000	0.552	—
HF hospitalization	14 (3.6)	5 (4.7)	32 (18.8)	<0.001	1.000	<0.001	—
Unstable angina hospitalization	49 (12.5)	20 (18.7)	31 (18.2)	0.008	0.124	0.012	—

MACCE, major adverse cardiovascular and cerebrovascular events; CV, cardiovascular; MI, myocardial infarction; HF, heart failure; Ph0, Phenotype 0 (Metabolically Balanced); Ph1, Phenotype 1 (Hyperglycemic-Dyslipidemic); Ph2, Phenotype 2 (Decompensated Inflammatory-Catabolic). Data are presented as *n* (%). *P* values were calculated using log-rank test for overall comparison and Bonferroni-corrected pairwise comparisons.

Analysis of individual MACCE components revealed that the between-phenotype differences were driven primarily by cardiovascular death (*P* < 0.001), non-fatal MI (*P* < 0.001), HF hospitalization (*P* < 0.001), and unstable angina hospitalization (*P* = 0.008). Non-fatal stroke (*P* = 0.089) and unplanned revascularization (*P* = 0.373) did not differ significantly across phenotypes. Cumulative incidence curves for each endpoint are presented in supplementary Figure S2.

### Independent prognostic value of cardiometabolic phenotype

**Figure 7:**
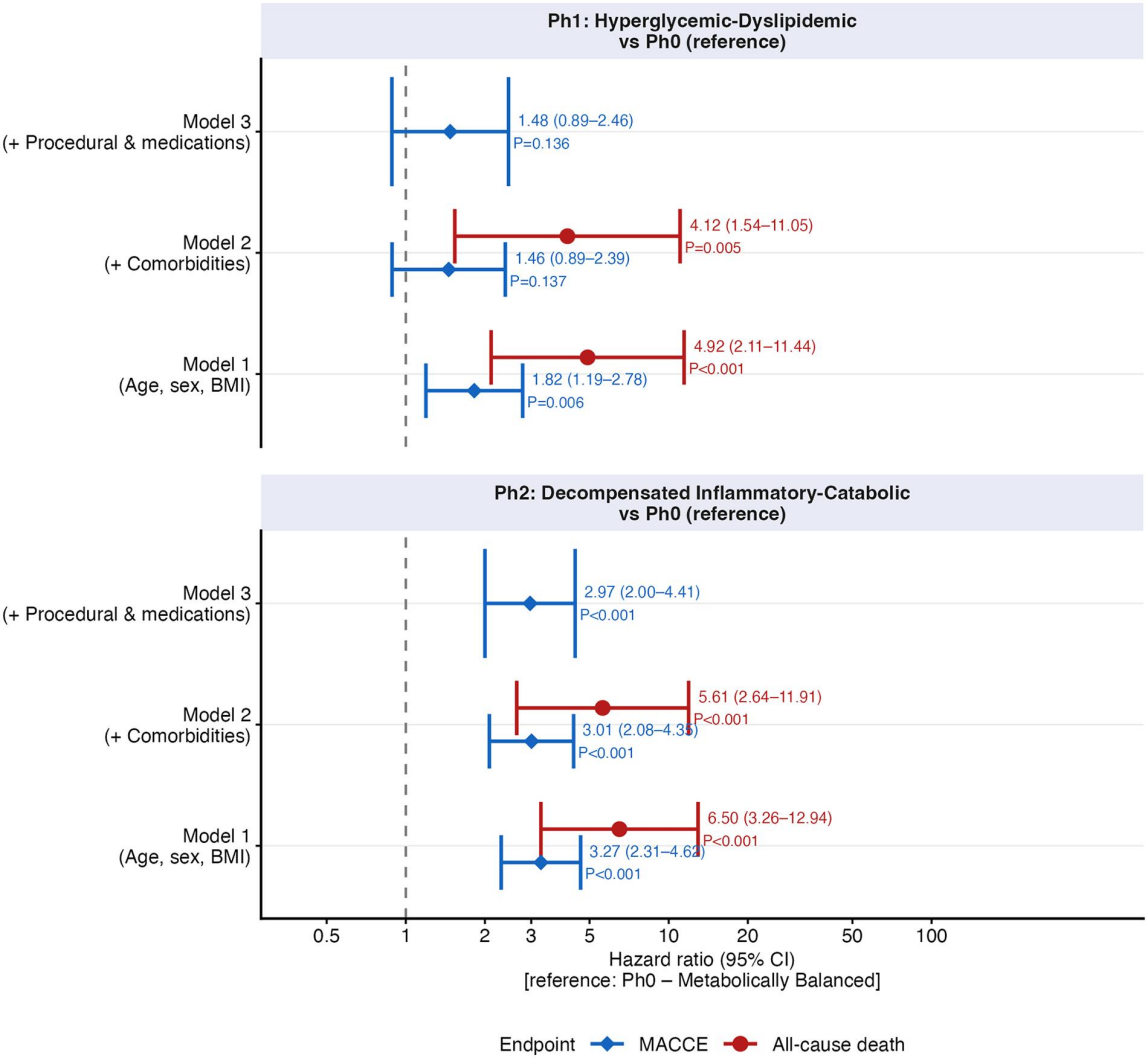
Multivariable Cox regression for MACCE and all-cause death. Hazard ratios (95% CI) for Ph1 and Ph2 relative to Ph0 (reference). Model 1: age, sex, BMI. Model 2: Model 1 plus smoking, hypertension, diabetes, dyslipidemia, prior MI, prior revascularization, prior stroke, and CKD. Model 3: Model 2 plus STEMI status, multivessel disease, implantation of ≥2 stents, admission GRACE score, beta-blockers, and PCSK9 inhibitors. All-cause death was modeled using Models 1 and 2 only owing to limited events (*n* = 61). BMI, body mass index; CI, confidence interval; CKD, chronic kidney disease; GRACE, global registry of acute coronary events; HR, hazard ratio; MI, myocardial infarction; PCSK9, proprotein convertase subtilisin/kexin type 9; STEMI, ST-elevation myocardial infarction.

Cox proportional hazards regression was used to evaluate the independent prognostic value of cardiometabolic phenotype for MACCE and all-cause death after progressive covariate adjustment (supplementary Table S3; Figure 7). For MACCE, Ph2 (Decompensated Inflammatory-Catabolic) consistently demonstrated significantly elevated risk relative to Ph0 across all three models: Model 1 adjusted for age, sex, and BMI (HR: 3.27, 95% CI: 2.31–4.62, *P* < 0.001), Model 2 additionally adjusted for comorbidities (HR: 3.01, 95% CI: 2.08–4.35, *P* < 0.001), and Model 3 further incorporating procedural variables and discharge medications (HR: 2.97, 95% CI: 2.00–4.41, *P* < 0.001). In contrast, Ph1 (Hyperglycemic-Dyslipidemic) was associated with a modestly increased MACCE risk in Model 1 (HR: 1.82, 95% CI: 1.19–2.78, *P* = 0.006), but this association was attenuated and no longer statistically significant after adjustment for comorbidities in Model 2 (HR: 1.46, 95% CI: 0.89–2.39, *P* = 0.137) or after full adjustment in Model 3 (HR: 1.48, 95% CI: 0.89–2.46, *P* = 0.136), suggesting that the excess MACCE risk observed in Ph1 is largely explained by the burden of comorbidities and treatment-related factors.

For all-cause death, Cox regression was restricted to Models 1 and 2, as the limited number of death events (*n* = 61) precluded stable estimation under the full covariate set of Model 3. Both phenotypes were associated with substantially higher mortality relative to Ph0. Ph2 showed a consistently strong association, with HR: 6.50 (95% CI: 3.26–12.94, *P* < 0.001) in Model 1 and HR: 5.61 (95% CI: 2.64–11.91, *P* < 0.001) in Model 2. Ph1 also showed elevated mortality risk, with HR: 4.92 (95% CI: 2.11–11.44, *P* < 0.001) in Model 1 and HR: 4.12 (95% CI: 1.54–11.05, *P* = 0.005) in Model 2; however, the wide confidence intervals reflect the limited number of events and these estimates should be interpreted with caution. The progressive attenuation of hazard ratios from Model 1 to Model 2 indicates that comorbidity burden partially accounts for the phenotype–mortality association, yet the associations remained statistically significant after adjustment.

### LDL-C stratification and phenotype-dependent risk

**Figure 8:**
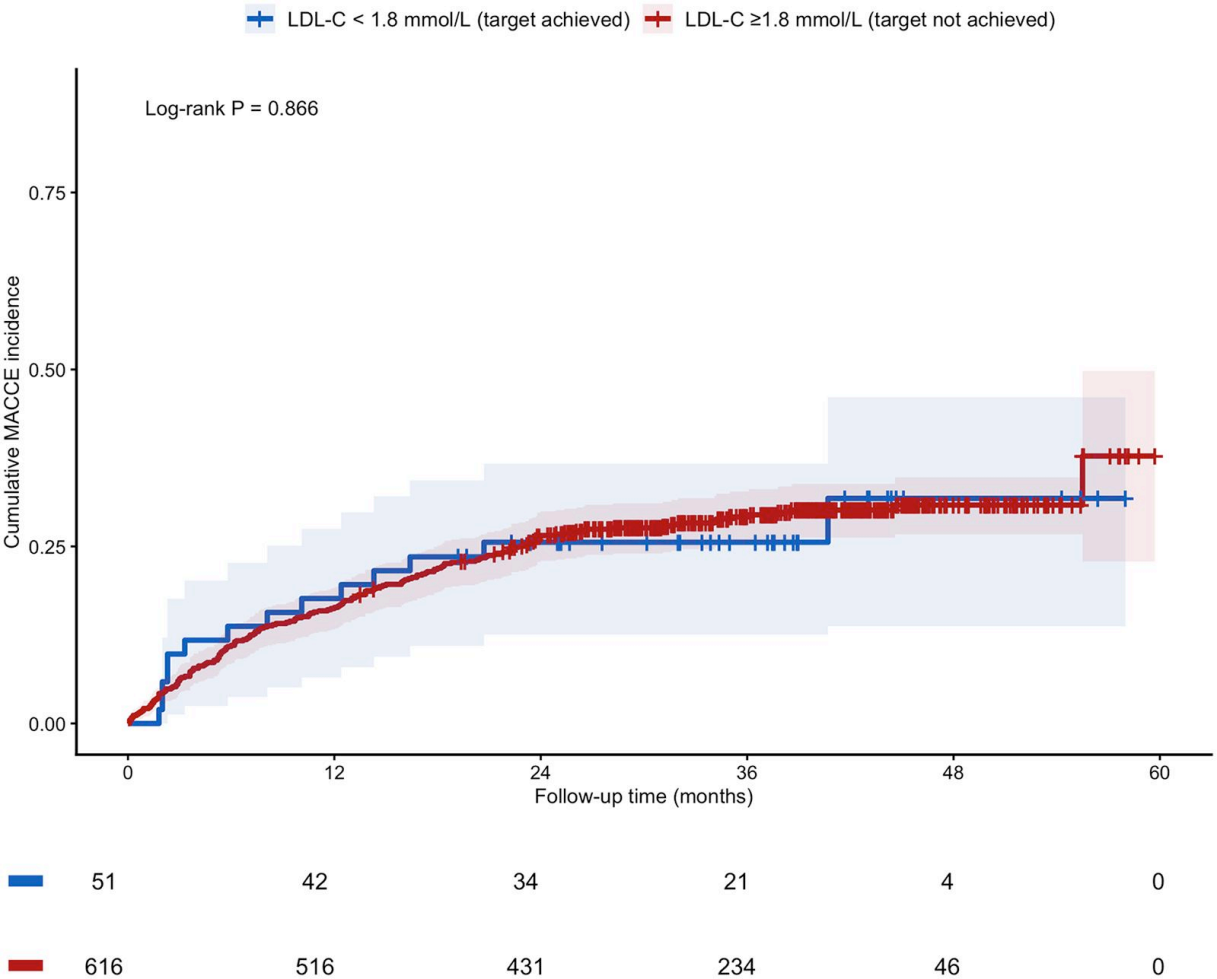
Cumulative MACCE incidence stratified by LDL-C target attainment. Kaplan–Meier curves comparing patients with LDL-C < 1.8 mmol/L vs. ≥1.8 mmol/L, corresponding to the ESC 2019 guideline target for very-high-risk patients. Numbers at risk are shown below the *x*-axis. *P* value from log-rank test. LDL-C, low-density lipoprotein cholesterol; MACCE, major adverse cardiovascular and cerebrovascular events.

**Figure 9:**
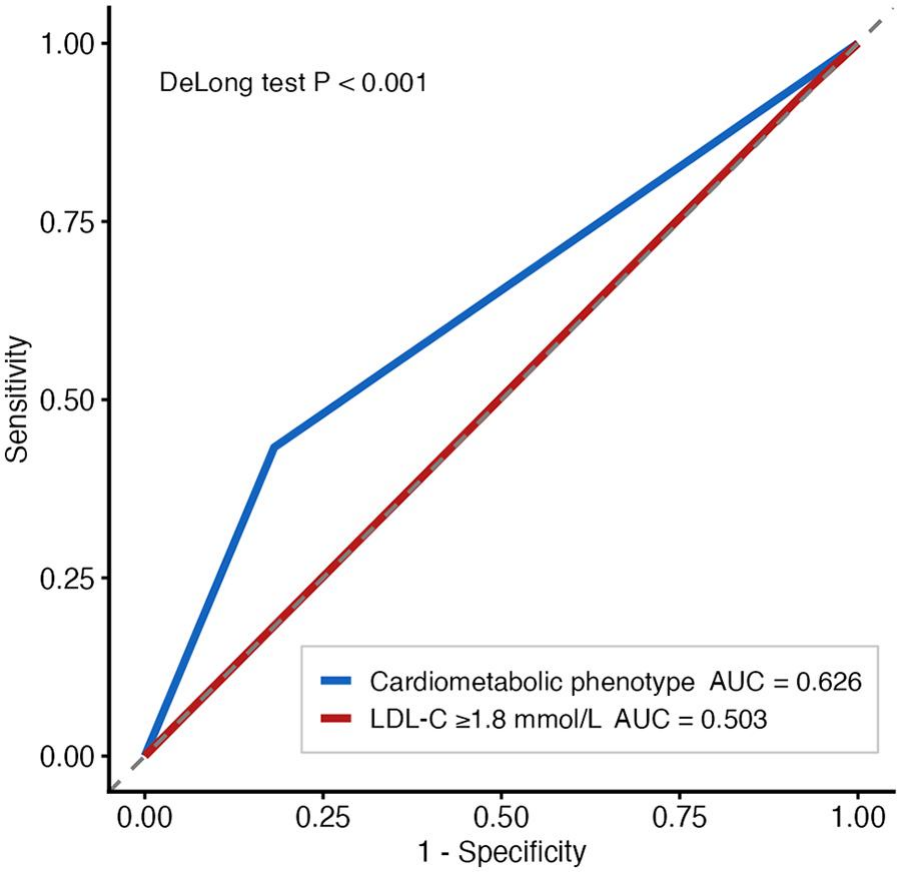
ROC curves comparing phenotype classification and LDL-C stratification for MACCE prediction. Cardiometabolic phenotype classification (Ph2 vs. Ph0 + Ph1) vs. LDL-C stratification (≥1.8 vs. < 1.8 mmol/L). AUC values with 95% confidence intervals are shown. *P* value from DeLong test. AUC, area under the curve; LDL-C, low-density lipoprotein cholesterol; MACCE, major adverse cardiovascular and cerebrovascular events; ROC, receiver operating characteristic.

A notable observation across the three phenotypes was the apparent dissociation between LDL-C levels and clinical outcomes. Despite having the lowest median LDL-C (2.695 mmol/L, IQR: 2.212–3.280), Ph2 exhibited the highest MACCE rate (49.4%) and all-cause mortality (20.6%), whereas Ph1—with the highest median LDL-C (3.260 mmol/L)—had intermediate event rates (supplementary Table S4). Stratification by LDL-C target attainment at the ESC 2019 threshold of 1.8 mmol/L did not discriminate MACCE risk (log-rank *P* = 0.866; Figure 8). Receiver operating characteristic analysis further confirmed the poor discriminative capacity of LDL-C–based classification (AUC 0.503), which was not significantly different from chance. In contrast, cardiometabolic phenotype classification yielded an AUC of 0.626 (95% CI: 0.587–0.665), and the difference between the two classifiers was statistically significant by DeLong testing (*P* < 0.001; Figure 9). Of note, only 51 patients had LDL-C < 1.8 mmol/L, which limits the power of the Kaplan–Meier comparison; however, the ROC analysis, which is not constrained by group size, corroborates this finding. It should be noted that phenotype classification was derived from unsupervised clustering rather than optimized as a predictive model; the comparison was therefore intended to demonstrate that metabolic phenotyping captures prognostic information beyond LDL-C stratification, not to propose it as a standalone risk score. Furthermore, the grouping of Ph2 vs. Ph0 + Ph1 for this ROC analysis reflects the *a priori* hypothesis of the study—that Ph2 represents a distinctly high-risk subgroup—established prior to outcome analysis rather than derived by inspecting outcome data. This conclusion is further corroborated by the Kaplan–Meier analysis, which requires no dichotomisation of phenotypes and similarly demonstrates no discriminative capacity for LDL-C target attainment (log-rank *P* = 0.866).

### Subgroup analysis

**Figure 10:**
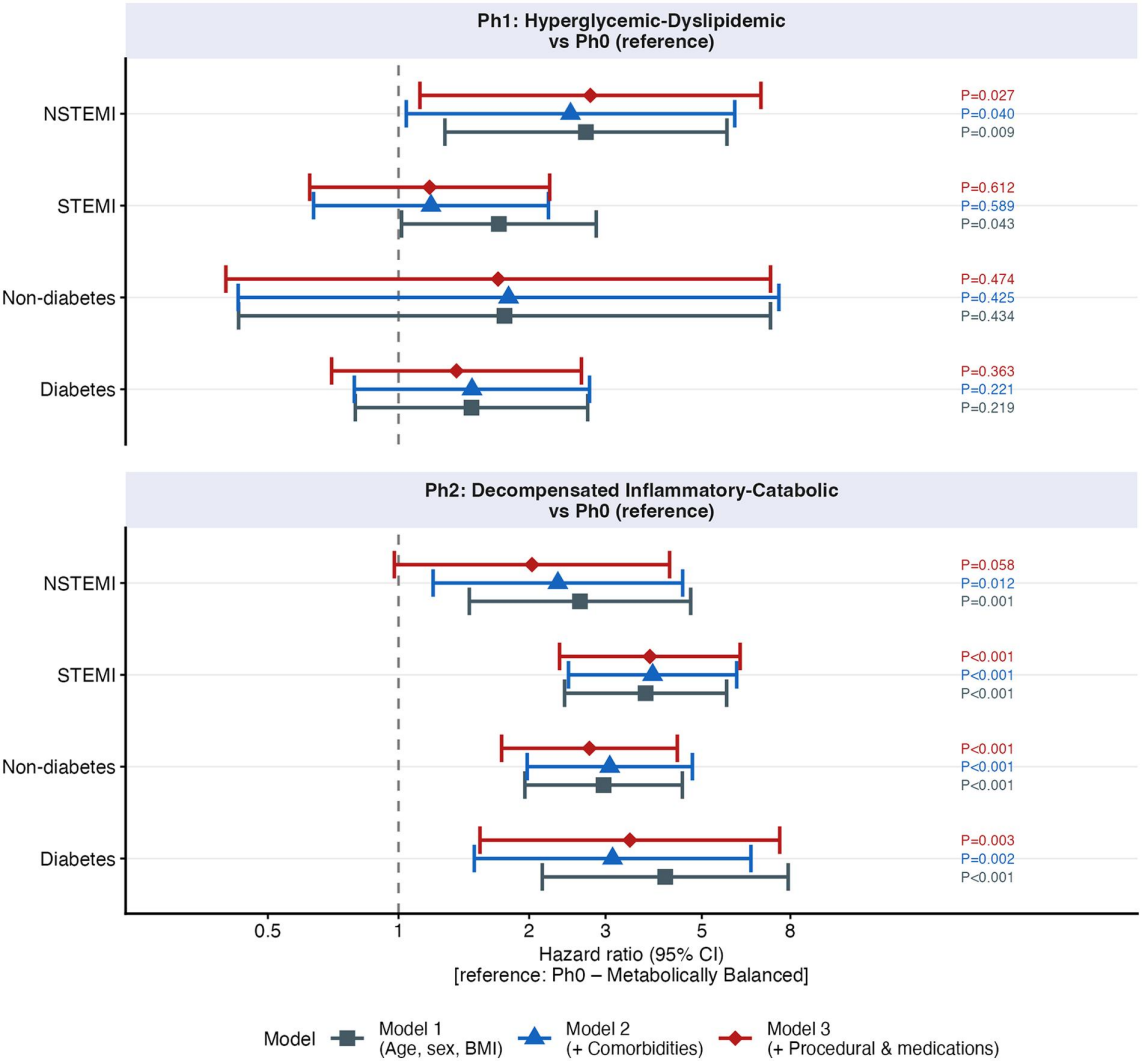
Subgroup Cox regression analyses for MACCE by phenotype. Hazard ratios (95% CI) for Ph1 and Ph2 relative to Ph0 across subgroups defined by diabetes status and AMI type. Model definitions as in Figure 7. AMI, acute myocardial infarction; CI, confidence interval; HR, hazard ratio; NSTEMI, non-ST-elevation myocardial infarction; STEMI, ST-elevation myocardial infarction.

To assess consistency of the prognostic associations across clinically relevant subgroups, Cox regression was repeated in patients stratified by AMI type and diabetes status (supplementary Table S5; Figure 10). Kaplan–Meier curves for MACCE across these subgroups are presented in supplementary Figure S3.

In the STEMI subgroup, Ph2 remained a robust independent predictor of MACCE across all three models (Model 1: HR: 3.71, 95% CI: 2.41–5.70; Model 2: HR: 3.85, 95% CI: 2.46–6.02; Model 3: HR: 3.80, 95% CI: 2.35–6.13; all *P* < 0.001). Ph1 was significantly associated with MACCE only in Model 1 (HR: 1.70, 95% CI: 1.02–2.86, *P* = 0.043), with the association attenuated after adjustment for comorbidities (Model 2: *P* = 0.589; Model 3: *P* = 0.612). In the NSTEMI subgroup, Ph1 showed a persistent association with MACCE across all three models (Model 1: HR: 2.70, 95% CI: 1.28–5.71, *P* = 0.009; Model 2: HR: 2.49, 95% CI: 1.04–5.95, *P* = 0.040; Model 3: HR: 2.77, 95% CI: 1.12–6.84, *P* = 0.027), whereas Ph2 was significant in Models 1 and 2 (Model 1: HR: 2.62, 95% CI: 1.46–4.71, *P* = 0.001; Model 2: HR: 2.33, 95% CI: 1.20–4.52, *P* = 0.012) but was attenuated to marginal non-significance in Model 3 (HR: 2.03, 95% CI: 0.98–4.21, *P* = 0.058).

In the non-diabetes subgroup, Ph2 was consistently associated with MACCE across all models (Model 1: HR: 2.97, 95% CI: 1.96–4.51; Model 2: HR: 3.07, 95% CI: 1.98–4.76; Model 3: HR: 2.75, 95% CI: 1.73–4.39; all *P* < 0.001), while Ph1 did not reach significance in any model, though the small number of non-diabetic Ph1 patients (*n* = 8) yielded wide confidence intervals that preclude firm conclusions. In the diabetes subgroup, Ph2 likewise retained significance across all models (Model 1: HR: 4.12, 95% CI: 2.14–7.91, *P* < 0.001; Model 2: HR: 3.11, 95% CI: 1.50–6.49, *P* = 0.002; Model 3: HR: 3.41, 95% CI: 1.54–7.56, *P* = 0.003), whereas Ph1 was not significantly associated with MACCE in any model.

### Sensitivity analysis

As a sensitivity analysis, the primary analyses were repeated in the 472 patients with observed HbA1c values (complete-case analysis). The progressive increase in cumulative MACCE incidence across phenotypes was preserved (overall log-rank *P* < 0.001; supplementary Figure S4), with pairwise comparisons confirming significant separation between Ph0 and both Ph1 (*P* = 0.006) and Ph2 (*P* < 0.001). In Cox regression, Ph2 remained independently associated with MACCE across all three models (Model 1: HR: 2.90, 95% CI: 1.90–4.43, *P* < 0.001; Model 2: HR: 2.26, 95% CI: 1.40–3.63, *P* < 0.001; Model 3: HR: 2.16, 95% CI: 1.31–3.55, *P* = 0.003). Ph1 likewise retained significance after full covariate adjustment (Model 1: HR: 2.02, 95% CI: 1.31–3.12, *P* = 0.002; Model 2: HR: 1.98, 95% CI: 1.19–3.29, *P* = 0.009; Model 3: HR: 2.03, 95% CI: 1.19–3.46, *P* = 0.010). These findings support the robustness of the phenotype–outcome associations to the HbA1c imputation strategy employed in the main analysis (supplementary Table S6). Of note, Ph1 achieved statistical significance across all three models in the complete-case analysis, whereas it was non-significant in Models 2 and 3 of the primary imputed analysis. One possible explanation is that patients with missing HbA1c—who were predominantly non-diabetic—were excluded from the complete-case cohort, resulting in a higher proportion of diabetic patients in whom the metabolic features defining Ph1 may carry greater discriminative weight. This finding should therefore be interpreted with caution, and the independent prognostic value of Ph1 requires further evaluation in larger cohorts.

## Discussion

Despite aggressive lipid-lowering therapy following acute myocardial infarction, substantial residual cardiovascular risk persists, and the drivers of this residual risk remain incompletely understood (19, 20). Using unsupervised clustering of routine cardiometabolic biomarkers, we identified a decompensated inflammatory-catabolic phenotype that independently predicted MACCE even after full covariate adjustment—and paradoxically, this highest-risk phenotype had the lowest LDL-C levels. Conventional LDL-C target attainment provided no prognostic discrimination, underscoring that residual risk in post-PCI AMI patients is driven by metabolic derangements beyond atherogenic lipoproteins. These findings highlight the potential of multi-dimensional phenotyping to identify high-risk subgroups who may require therapeutic strategies beyond lipid-lowering.

Data-driven phenotyping has gained traction in cardiovascular medicine since Shah et al. applied unsupervised clustering to HFpEF and identified three prognostically distinct phenogroups (21), an approach subsequently extended to cardiogenic shock (22) and MI complicated by cardiac arrest (23). In the AMI setting, D'Ascenzo et al. clustered 23,270 ACS patients using demographic and procedural variables (24), and Shetty et al. identified four phenogroups in 470,960 STEMI patients from the US National Inpatient Sample (18). Our study differs from these prior efforts in that we restricted clustering inputs to cardiometabolic biomarkers—all obtainable from routine admission blood tests—and evaluated outcomes over a median follow-up exceeding 30 months, testing whether metabolic phenotyping captures residual prognostic heterogeneity beyond what conventional risk factors address.

The most striking finding is the identification of Ph2, a decompensated inflammatory-catabolic phenotype characterized by the convergence of three pathophysiological processes: systemic inflammation (elevated CRP), nutritional depletion (low albumin, low hemoglobin), and cardiac decompensation (depressed LVEF, elevated NT-proBNP). While the prognostic relevance of each individual marker is well established, what our data reveal is that these abnormalities co-occur within a recognizable patient subgroup, suggesting shared underlying pathophysiology rather than independent risk factors. Importantly, Ph2 remained independently associated with MACCE even after full adjustment for age, sex, BMI, major comorbidities, and the GRACE score (Model 3: HR: 2.97, *P* < 0.001), indicating that this phenotypic construct captures risk beyond what is explained by conventional clinical variables.

The attenuation of Ph1 after comorbidity adjustment reflects substantial overlap between this phenotype and diabetes: 92.5% of Ph1 patients had diabetes, and the metabolic features defining Ph1—severe hyperglycemia and atherogenic dyslipidemia—are hallmark complications of diabetes. In the overall cohort, once diabetes is entered into the model, the phenotype label adds little discriminative information for MACCE beyond known diabetes status. Intriguingly, this pattern does not hold uniformly across AMI subtypes. In NSTEMI, Ph1 remained independently associated with MACCE across all models (Model 3: HR: 2.77, *P* = 0.027), whereas in STEMI, its association was attenuated to non-significance after comorbidity adjustment. This divergence can be understood through the distinct pathophysiological profiles of these two conditions. STEMI arises from sudden, complete coronary occlusion, typically following acute plaque rupture (25). In this setting, outcomes are dominated by the severity of the acute insult—infarct size (26), hemodynamic compromise (27), and the intensity of the acute-phase inflammatory response. These overwhelming acute factors effectively eclipse the prognostic contribution of chronic metabolic abnormalities. Indeed, after adjusting for the GRACE score—which heavily weights acute hemodynamic parameters such as blood pressure, heart rate, and renal function at presentation (28)—the Ph1 classification adds little incremental prognostic value. The chronic hyperglycemia and dyslipidemia defining Ph1, while pathogenic over years, become less relevant in determining short- to medium-term outcomes when the primary determinant is the magnitude of acute myocardial necrosis. In contrast, NSTEMI typically results from partial vessel occlusion and unfolds in patients with more extensive multivessel coronary disease (29). Here, prognosis is less tethered to the severity of the index event and more dependent on the patient's overall atherosclerotic burden and ongoing metabolic dysfunction (30, 31). The persistent significance of Ph1 in NSTEMI—even after full covariate adjustment—suggests that the chronic metabolic dysregulation it represents continues to fuel atherothrombotic risk through mechanisms not fully captured by traditional comorbidity measures. This interpretation is supported by studies demonstrating that glucose and other metabolic biomarkers have stronger prognostic value in NSTEMI than in STEMI (32), and that NSTEMI patients harbor more diffuse coronary atherosclerosis (29, 33), making them vulnerable to recurrent events driven by disease progression in non-culprit territories.

The dissociation between LDL-C levels and outcomes in our cohort is consistent with the “lipid paradox” reported across multiple AMI populations. Cho et al. (7)and Reddy et al. (34) independently demonstrated an inverse association between admission LDL-C and mortality in large MI registries. Zeng et al. showed that this paradox was confined to patients with high inflammatory burden (hsCRP ≥ 3 mg/L) (9), and Lu et al. demonstrated it was predominantly observed in malnourished patients (10)—a profile closely mirroring our Ph2. Han et al. further confirmed that the paradox is substantially attenuated after adjusting for frailty, inflammation, and malnutrition (35), and Wulff et al. proposed that larger infarctions cause greater acute-phase LDL-C consumption for tissue repair (36). Our findings add a phenotypic dimension to this literature: the low LDL-C in Ph2 co-occurs with markers of systemic catabolism, suggesting it reflects disease severity rather than effective lipid management. This does not argue against LDL-C lowering therapy, but it does suggest that LDL-C alone is insufficient for residual risk assessment in post-PCI AMI patients.

Ph2 identifies a subgroup carrying disproportionately high residual risk in whom the dominant risk drivers appear to be inflammation and nutritional depletion rather than atherogenic lipid burden. The CANTOS (37), COLCOT (38), and LoDoCo2 (39) trials have demonstrated the cardiovascular benefit of anti-inflammatory therapy in patients with coronary disease, while Raposeiras Roubin et al. (40)highlighted the independent prognostic impact of malnutrition in 5,062 ACS patients. Whether anti-inflammatory or nutritional interventions would disproportionately benefit Ph2-like patients is an open question warranting prospective investigation. The phenotypic framework presented here could help identify such candidates, though external validation is needed before clinical application.

Several methodological points merit discussion. The Silhouette score (0.1648) was modest, reflecting the continuous nature of cardiometabolic variation in this population—a pattern that is typical of clinical phenotyping studies in complex cardiovascular disease, where biological data rarely achieve the geometric separation seen in synthetic datasets. Critically, a low Silhouette score does not preclude clinical validity: the most meaningful test of a phenotyping solution is whether it identifies groups with meaningfully different outcomes. In this regard, the three phenotypes demonstrated a consistent and graded prognostic gradient, with MACCE rates of 19.4%, 31.8%, and 49.4% across Ph0, Ph1, and Ph2, respectively, and significant pairwise separation across all Kaplan–Meier comparisons (all *P* < 0.01). Ph2 remained independently associated with MACCE after full covariate adjustment (HR: 2.97, 95% CI: 2.00–4.41, *P* < 0.001), confirming that the cluster boundaries, modest as they are geometrically, capture real and clinically consequential heterogeneity. Algorithmic robustness was further supported by near-perfect agreement with PAM (ARI = 0.997) and stable bootstrap distributions (mean Silhouette 0.1694, 95% CI: 0.1376–0.2081). The moderate GMM agreement (ARI = 0.308) reflects the methodological difference between hard and probabilistic assignment.

We intentionally excluded clustering variables (LVEF, NT-proBNP, CRP, albumin, creatinine) from the Cox models to avoid overadjustment bias—including them would adjust away the very construct being evaluated, as the phenotype is itself derived from these variables. A related question is whether Ph2 merely reflects the additive effect of its constituent biomarkers measured individually. We would argue that the value of unsupervised phenotyping is not to identify new biomarkers, but to identify patients in whom multiple abnormalities converge simultaneously. In routine clinical practice, a patient with moderately elevated CRP, moderately reduced albumin, and moderately depressed LVEF would not trigger concern on any individual measure, yet the simultaneous co-occurrence of these derangements defines Ph2 and confers substantially elevated risk. This convergent pathophysiological pattern suggests a shared underlying disease state that individual biomarkers, evaluated independently or additively, fail to capture. Formally demonstrating incremental prognostic value over a composite biomarker panel nonetheless requires prospective evaluation with pre-specified biomarker adjustment, which we identify as a priority for future research.

The limited number of death events (*n* = 61) precluded fitting Model 3 for all-cause mortality and resulted in wide confidence intervals for the mortality estimates, particularly for Ph1. These mortality findings should be regarded as hypothesis-generating. With 61 death events and 11 covariates in Model 2, the events-per-variable ratio was approximately 5.5, which is below the conventionally recommended threshold of 10; accordingly, these mortality estimates should be regarded as exploratory and interpreted with caution.

### Limitations

This study has several limitations. First, the retrospective single-centre design limits generalisability, and prospective external validation in independent cohorts is needed before clinical application. Second, HbA1c was missing in 16.5% of patients, predominantly among non-diabetic individuals, suggesting a missing-not-at-random pattern. Although multiple imputation with diabetes status as an auxiliary variable was employed to partially mitigate this bias, residual bias cannot be fully excluded. Notably, Ph1 showed discrepant significance between the primary imputed and complete-case analyses, and its independent prognostic value therefore remains uncertain pending prospective validation in larger cohorts with systematic HbA1c ascertainment. Third, LDL-C was dichotomized at 1.8 mmol/L rather than the current ESC guideline-recommended target of <1.4 mmol/L for very-high-risk patients, as only a minority achieved the more stringent threshold—specifically, only 51 patients (7.6%) achieved LDL-C < 1.8 mmol/L, and applying the 1.4 mmol/L threshold would reduce the attainment group to approximately 5%–10% of patients, precluding meaningful comparison. The 1.8 mmol/L cutoff, while providing adequate power for analysis, may not fully capture contemporary treatment targets. Additionally, LDL-C was measured as a single admission value and does not reflect longitudinal lipid exposure or treatment target attainment during follow-up; the prognostic comparison should therefore be interpreted in the context of point-of-care lipid assessment rather than optimised lipid management. Fourth, the relatively small number of death events (*n* = 61) precluded full Cox adjustment for all-cause mortality and resulted in wide confidence intervals, particularly for Ph1. Fifth, residual confounding from unmeasured variables—including medication adherence, lifestyle modification, and longitudinal metabolic changes—cannot be ruled out despite progressive covariate adjustment.

## Conclusion

In this cohort of 668 post-PCI AMI patients, unsupervised K-means clustering of 13 routine cardiometabolic variables identified three phenotypes with distinct metabolic profiles and divergent clinical trajectories. The decompensated inflammatory-catabolic phenotype (Ph2) was independently associated with MACCE after full covariate adjustment and carried the highest event rate despite having the lowest LDL-C, while conventional LDL-C target attainment at the 1.8 mmol/L threshold provided no prognostic discrimination. These findings indicate that residual cardiovascular risk in post-PCI AMI patients is driven by multi-dimensional metabolic derangements—encompassing inflammation, nutritional depletion, and cardiac decompensation—that LDL-C alone fails to capture. Multi-dimensional cardiometabolic phenotyping may offer a complementary framework for identifying high-risk subgroups who could benefit from targeted interventions beyond conventional lipid-lowering therapy.

## Data Availability

The raw data supporting the conclusions of this article will be made available by the authors, without undue reservation.
